# Impact of Silicone Oil Removal on Macular Perfusion

**DOI:** 10.3390/tomography8040146

**Published:** 2022-07-06

**Authors:** Michelle Prasuhn, Felix Rommel, Armin Mohi, Salvatore Grisanti, Mahdy Ranjbar

**Affiliations:** Department of Ophthalmology, University Hospital Schleswig-Holstein, University of Luebeck, Ratzeburger Allee 160, 23538 Luebeck, Germany; felix.rommel@uksh.de (F.R.); armin.mohi@uksh.de (A.M.); salvatore.grisanti@uksh.de (S.G.); eye.research101@gmail.com (M.R.)

**Keywords:** silicone oil endotamponade, optical coherence tomography angiography, retinal detachment, macular perfusion

## Abstract

(1) Background: Silicone oil (SO) can be used as an endotamponade during vitreoretinal surgery for retinal detachment. There is emerging evidence that SO filling of the vitreous cavity and its removal may impact macular perfusion. So far, studies have not focused on choroidal sublayer perfusion, yet. (2) Methods: Optical coherence tomography angiography was applied in 19 patients with SO endotamponade before and four weeks after removal of SO. (3) Results: Perfusion of choriocapillaris increased significantly after SO removal, while perfusion of Haller’s and Sattler’s layer decreased significantly. (4) Conclusions: Removal of SO impacts choroidal perfusion and leads to a perfusion shift within choroidal sublayers. This study underlines that it is worth to conduct larger prospective studies that evaluate the choroidal perfusion and its functional implications in more detail.

## 1. Introduction

Silicone oil (SO) is commonly used as endotamponade in vitreoretinal surgery for retinal detachment. It is mainly reserved for rather complicated cases as complications due to SO can occur. Most commonly, this includes cataract induction, increased intraocular pressure (IOP), emulsification of SO, or keratopathy [1]. A feared complication, which is not fully understood to date, is severe deterioration of central vision even in uncomplicated cases with preoperatively seemingly good visual prognosis [2,3,4]. However, SO is an important addition to the operative repertoire of vitreoretinal surgeons and is a reasonable choice in many cases.

Endotamponade with SO, as well as its removal from the vitreous cavity are known to impact retinal and choroidal microstructures [5]. Several studies with different methodological approaches attempted to examine vascular and perfusion alterations due to SO tamponade and its removal [6,7,8]. Optical coherence tomography angiography (OCTA) is a non-invasive vascular imaging technique that allows us to look at this question more in detail. Even though it has several drawbacks like artifacts or imaging a limited area of the retina, it can be used in research to evaluate microvasculature changes of retina and choroid [9]. The perfusion of the retina and choroid, in turn, can impact functional parameters like visual acuity, which also makes it interesting in a clinical context.

The aim of this study was to evaluate how retinal and choroidal perfusion change due to SO removal with particular emphasis on choroidal sublayers.

## 2. Materials and Methods

Participants for this retrospective study were enrolled at the Department of Ophthalmology at the University of Lübeck between April 2021 and March 2022. The study was approved by the institutional review board and was conducted in accordance with the Declaration of Helsinki. Written informed consent was obtained individually before enrolment. Inclusion criteria included (1) silicone oil endotamponade after vitrectomy due to rhegmatogenous retinal detachment; (2) indication for SO removal; (3) pseudophakia. Exclusion criteria were: (1) evidence or history of systemic disorders, including cardiovascular disease, antihypertensive drug use and diabetes mellitus; (2) history of other ocular surgeries other than uncomplicated cataract surgery; (3) macular edema; (4) indication for epiretinal membrane peeling and internal limiting membrane peeling during surgery for retinal detachment and SO removal.

Surgery for retinal detachment and silicone oil removal were performed by the same vitreoretinal surgeons (M.R., F.R., A.M.), applying the same clinical standards. For retinal detachment surgery, all patients underwent vitrectomy via 23-gauge trocars that were placed 3.5 mm away from the limbus. Perfluorocarbon liquid was installed for retinal reattachment and all patients received 360° endolaser coagulation. Silicone oil was chosen as these patients showed rather complex cases, including giant retinal tears, proliferative vitreoretinopathy, and multiple retinal breaks. All patients received silicone oil with the same characteristics (5000 centistokes, DORC, Zuidland, The Netherlands).

At baseline, all participants underwent an examination including refraction, best-corrected visual acuity (BCVA) in Snellen, axial length measurement, slit-lamp biomicroscopy and IOP. The maximum permissible spherical and cylindrical aberration was ±3 and ±1 diopters, respectively. Only patients with an IOP within the normal range (10–21 mmHg) were included. Imaging was performed at baseline and four weeks after SO removal with prior pupil dilatation using the Zeiss Cirrus HD-OCT (AngioPlex, CIRRUS HD-OCT model 5000, Carl Zeiss Meditec, Inc., Dublin, CA, USA) by a single, trained operator (M.P.). The devices’ follow-up mode was used to assure measurements at the same location for both time points. Each imaging session included Enhanced Depth Imaging (EDI)-OCT scans (10 × 10 mm^2^) and OCTA volumetric scans (6 × 6 mm^2^) of the posterior pole. OCTA scans with a signal strength ≥7 and without motion, segmentation or projection artifacts were utilized to avoid misinterpretation [10]. To avoid bias due to physiological diurnal changes of the ocular perfusion, all examinations were carried out around noon [11,12] Total macular volume (TMV) was automatically calculated by the device. Subfoveal choroidal thickness (SFCT) was measured perpendicularly below the fovea by two experienced graders (F.R. and M.P.) and the obtained values were averaged for further analysis. OCTA images were automatically segmented in all B-scans according to the manufacturer’s default setting to produce en face images of full retina (FR) slabs. Manual segmentation (M.P.) was performed to get 20 µm slabs of the choriocapillaris (CC), Sattler’s layer (SL) and Haller’s layer according to previously published protocols [10,12]. Each acquired en face image was exported into ImageJ (NIH, Version 1.52e, Bethesda, Rockville, MD, USA) and binarized using the Otsu method, an automatic threshold selection from gray-level histograms in order to determine the percentage of white and black pixels [13]. As in previous publications, FR perfusion (FRP) and CC perfusion (CCP) were calculated by recording the percentage of white pixels, while for SL perfusion (SLP) and HL perfusion (HLP) black pixels were considered [10,11,12].

Statistics were performed using Prism GraphPad (version 8.0, La Jolla, CA, USA). BCVA in decimal Snellen was converted to the logarithm of the minimum angle of resolution (logMAR). The Shapiro–Wilk test was used to check for normality of the obtained data. Except for BCVA and TMV, the data were found to be normally distributed, so a paired *t*-test was used to compare baseline and follow-up values of the same eye. For TMV and BCVA, a Wilcoxon test was used. Possible interactions between the morphological and functional parameters were analyzed using Pearson’s correlation analysis. A *p*-value of <0.05 was considered statistically significant.

## 3. Results

A total of 19 eyes from 19 patients were enrolled in this study. Demographic and clinical data are reported in Table 1. Twelve (63.2%) male and seven (36.8%) female participants were included in this study, with a mean age of 62.3 years. About two thirds of patients had a detached macula when the eye was filled with SO.

Table 2 summarizes changes in BCVA, morphological data as seen on OCT, as well as perfusion values. Silicone oil removal resulted in a significant increase in BCVA. Morphologically, we noticed an increase in CRT and TMV. It can be seen that choroidal perfusion changed due to SO removal: While CCP increased significantly, we detected a significant decrease of SLP and HLP. At the same time, SFCT remained steady. Perfusion values of the retina did not change due to SO removal.

Functional and morphological data were correlated at baseline and after four weeks as summarized in Table 3 and Table 4. The duration of SO endotamponade correlated with the macular status (whether macula was on or off before SO). Macular status also correlated with BCVA, at baseline as well as after SO removal.

## 4. Discussion

This retrospective OCTA study analyzed perfusion changes due to SO removal. To our knowledge, this is the first study to focus on perfusion of the choroid and its sublayers in this regard.

Previous studies that analyzed perioperative perfusion suggested that SO endotamponade and its removal impact the microvasculature. For example, Effert at al. noticed a prolonged arteriovenous passage time in eyes filled with SO compared to the fellow eye [7]. Kubicka et al. showed long term effects on the retinal microperfusion using Heidelberg Retina Flowmeter [8]. Consequently, OCTA studies were conducted. So far, OCTA based studies mainly evaluated the impact of SO filling, in which alterations of the microvasculature were present [6,14,15]. However, data on the impact of SO removal is limited, and studies on choroidal changes in this context do not exist as far as we know [16,17]. An OCTA study by Bayraktar et al. evaluated the superficial and deep capillary plexus of the retina before and after SO removal and compared patients with attached and detached macula before vitreoretinal surgery [17]. Removal of SO resulted in permanent morphological and vascular alterations as compared with the healthy fellow eyes, which did not disappear after SO removal in the group of eyes with macula-off retinal detachment. The study also showed transient alterations as compared with their fellow eyes in the group of eyes with macula-on retinal detachment before SO removal, but all of them improved and returned almost normally following SO removal.

In our OCTA study, we noticed a significant increase in CCP and a decrease in HLP and SLP due to SO removal. These findings are of high interest, as so far there was no evidence that SO removal impacts choroidal sublayer perfusion. Underlying pathomechanisms for this perfusion shift, however, remain elusive and can only be speculated about. The choroid primarily consists of vessels that can be subdivided into small vessels in the CC, medium-sized vessels in SL, and large vessels in HL. There might be a mechanical component due to the SO that could compress the adjacent CC. This physical pressure on the CC is eliminated after SO removal so that CCP increases with consecutive decrease of HLP and SLP. Interestingly, these changes do not have any significant impact on SFCT. Choroidal thickness data of studies focusing on SO removal are quite controversial. Karimi et al. showed that SFCT was reduced in patients receiving SO tamponade, which did not change after SO removal. However, a study by Mirza et al. reported a decrease in SFCT after removal of SO [18]. The choroid is the main oxygen supplier of the retina and plays an important role in maintaining outer retinal layers, and pathological changes in its circulation may lead to consecutive alterations of the retinal pigment epithelium and retinal substructures [19]. Successively, visual function can also be dependent on choroid, so this substructure should not be neglected when studying morphological alterations. Our data did not allow to determine how choroidal perfusion impacts the visual outcome; this would be an interesting primary outcome measure for future studies. The exact role of the choroidal sublayers is not fully understood. The CC is formed by fenestrated capillaries arranged in one thin sheet plane. The capillaries are arranged into a sequence of small fenestrated lobules. It is characterized by a high flow rate in the macular area with fewer intravascular space in order to provide oxygen and nutrients, and remove the metabolic waste products from the RPE and photoreceptors [19,20]. Haller’s and Sattler’s layer are located below the CC. The Sattler’s layer consists of medium sized vessels, and the Haller’s layer of larger sized vessels. Their roles are complex and exact mechanisms still unclear. Choroidal thickness- and perfusion changes are speculated to be caused by changes in the synthesis of osmotically active molecules, by changes in vascular permeability, changes in muscle tonus, or even flux from the anterior chamber or along the RPE [19]. Changes in the choroidal sublayers can be seen in various ocular and systemic diseases, and we are starting to gain more and more information on its role in these conditions [9].

Expectedly, our data showed that a detached macula is associated with a poorer BCVA, which can be seen at baseline, as well as four weeks after SO removal. These findings are in accordance with previous studies, in which a detached macula was identified as one of the most important prognostic factors for future visual function [3,21]. According to our data, the duration of SO endotamponade did not impact the visual outcome. Previous studies have reported similar results [4,22]. However, our cohort had a relatively homogeneous time frame, in which the SO remained in the patients’ vitreous cavities, ranging from six to fifteen weeks, which limits the significance of these results. Macula off status was associated with a longer duration until SO was removed.

Our data on central retinal thickness changes were also in keeping with previous data, that have been reviewed by Ghanbari et al.: While central macular thickness reduces due to SO filling after retinal detachment repair, this effect is reversible, and CRT increases after SO removal [5]. Our data support this as CRT increases significantly after SO removal in our cohort. Central retinal thinning might be caused by various factors like mechanical stress on the inner retinal layers, inflammatory mechanisms, toxicity on the internal limiting membrane or hydrophobicity of the oil that alters the retinal environment. Luckily, these effects seem to be short term and reversible to a large extent and is dependent on the duration of SO filling [5].

In our study group, all patients received 5000 centistokes SO which is characterized by a high viscosity. It is so far unclear, to what extent the viscosity plays a role in retinal and choroidal perfusion. Our study design did not aim at evaluating this in detail, but future studies could compare different SO endotamponades to gain further insights.

The main limitation of our study is its retrospective character and the relatively small study population. Therefore, more detailed analyses like regression models could not be done and analyzing group differences was hampered. Studying our cohort with a baseline measurement and only one more measurement after four weeks gives a good insight into certain mechanisms, however, dynamic processes might be overlooked. For example, short term changes before and after silicone oil filling, as well as removal, might be interesting for future studies. Also, longer-term follow-up visits might reveal changes in perfusion that differ from the point of time that we chose. Moreover, the understanding and interpretation of various signals in the choroidal vasculature on OCTA analysis are controversial and need further research. Possible gender-specific differences regarding ocular perfusion changes were not taken into account when recruiting participants, resulting in an unbalanced gender ratio. Potential influencing factors on OCTA analysis such as hematocrit, caffeine, and hormone status were not recorded but are expected to be of limited significance.

However, we were able to gain first insights into perfusion changes of the choroid due to SO removal. It would be of interest to look at the exact implications more in depth with larger prospective studies, especially focusing on functional parameters.

## 5. Conclusions

In conclusion, we provide the first study to find a significant change in choroidal perfusion due to SO removal. An increase of CCP with concomitant decrease of HLP and SLP while SFCT remains steady give first insights into choroidal mechanisms when the mechanical pressure of the SO is being removed from the vitreous cavity. This study is a door opener for larger prospective studies that should focus on the implications of this choroidal perfusion shift on functional, and therefore clinically even more relevant parameters.

## Figures and Tables

**Table 1:** Demographic and clinical characteristics of the study cohort. Data are presented as mean ± standard deviation.

Parameter	Mean
Gender (male/female), *n* (%)	12/7 (63.2/36.8)
Age (years)	62.3 ± 9.65
Duration of oil tamponade (weeks)	10.0 ± 2.7
Laterality (right/left), *n* (%)	9/10 (47.37/52.63)
Initial status macula (on/off), *n* (%)	6/13 (31.6/68.4)

**Table 2:** Comparison of best-corrected visual acuity (BCVA), anatomical and functional parameters before and 4 weeks after silicone oil removal.

Parameter	Baseline	4 Weeks	*p*-Value
BCVA (logMAR)	0.5 (0.2; 1.3)	0.4 (0.0; 1.0)	<0.0001 (Wilcoxon)
CRT (µm)	243 ± 40.8	263 ± 32.1	0.0047
TMV (mm^3^)	9.8 (8.9; 11.9)	10.2 (9.4; 12.2)	<0.0001 (Wilcoxon)
SFCT (µm)	276 ± 89.6	284 ± 102	0.434
FRP (%)	26.51 ± 5.2	27.39 ± 6.7	0.596
CCP (%)	45.09 ± 3.0	45.8 ± 2.9	0.0013
SLP (%)	59.19 ± 2.4	57.72 ± 3.6	0.034
HLP (%)	62.37 ± 6.3	60.53 ± 4.8	0.0402

*p*-values were calculated with a paired *t*-test for normally distributed parameters and a Wilcoxon test for non-normally distributed data. BCVA: best-corrected visual acuity; CCP: choriocapillaris perfusion; CRT: central retinal thickness, duration: duration of silicone oil tamponade; FRP: full retinal perfusion; on/off: macular status preoperatively (whether macula was attached or detached); HLP: Haller’s layer perfusion; SFCT: subfoveal choroidal thickness; SLP: Sattler’s layer perfusion; TMV: total macular volume.

**Table 3:** Correlation analysis of functional and morphological data at baseline.

Parameter		Duration	on/off	BCVA	CRT	SFCT	FRP	CCP	HLP	SLP
duration	CC	1	−0.656	−0.199	0.316	−0.091	−0.039	−0.201	−0.053	0.572
	*p*	0	0.002	0.415	0.188	0.711	0.874	0.410	0.828	0.011
on/off	CC	−0.656	1	0.513	−0.085	−0.059	0.316	−0.070	0.104	−0.351
	*p*	0.002	0	0.046	0.731	0.809	0.187	0.775	0.671	0.140
BCVA	CC	−0.199	0.014	1	−0.410	0.279	−0.153	−0.288	−0.048	0.212
	*p*	0.415	0.956	0	0.081	0.247	0.531	0.231	0.847	0.384
CRT	CC	0.316	−0.085	−0.410	1	−0.081	0.339	0.108	−0.275	0.074
	*p*	0.188	0.731	0.081	0	0.741	0.156	0.660	0.255	0.762
SFCT	CC	−0.091	−0.059	0.279	−0.081	1	−0.239	0.148	0.098	0.022
	*p*	0.711	0.809	0.247	0.741	0	0.324	0.546	0.690	0.930
FRP	CC	−0.039	0.316	−0.153	0.339	−0.239	1	−0.221	−0.183	0.084
	*p*	0.874	0.187	0.531	0.156	0.324	0	0.363	0.454	0.731
CCP	CC	−0.201	−0.070	−0.288	0.108	0.148	−0.221	1	−0.013	−0.433
	*p*	0.410	0.775	0.231	0.660	0.546	0.363	0	0.958	0.064
HLP	CC	−0.053	0.104	−0.048	−0.275	0.098	−0.183	−0.013	1	0.377
	*p*	0.828	0.671	0.847	0.255	0.690	0.454	0.958	0	0.111
SLP	CC	0.572	−0.351	0.212	0.074	0.022	0.084	−0.433	0.377	1
	*p*	0.011	0.140	0.384	0.762	0.930	0.731	0.064	0.111	0

BCVA: best-corrected visual acuity; CC: correlation coefficient; CCP: choriocapillaris perfusion; CRT: central retinal thickness, duration: duration of silicone oil tamponade; FRP: full retinal perfusion; on/off: macular status preoperatively (whether macula was attached or detached); HLP: Haller’s layer perfusion; *p*: *p*-value; SFCT: subfoveal choroidal thickness; SLP: Sattler’s layer perfusion; TMV: total macular volume.

**Table 4:** Correlation analysis of functional and morphological data four weeks after silicone oil removal.

Parameter		Duration	on/off	BCVA	CRT	SFCT	FRP	CCP	HLP	SLP
duration	CC	1	−0.656	−0.334	0.276	−0.072	−0.372	−0.118	0.221	0.440
	*p*	0	0.002	0.163	0.252	0.769	0.117	0.632	0.362	0.059
on/off	CC	−0.656	1	0.467	−0.024	0.070	0.381	−0.084	0.000	−0.257
	*p*	0.002	0	0.044	0.921	0.775	0.108	0.732	1.000	0.289
BCVA	CC	−0.334	0.467	1	−0.386	0.042	0.112	−0.389	−0.024	−0.252
	*p*	0.163	0.044	0	0.102	0.864	0.647	0.100	0.921	0.298
CRT	CC	0.276	−0.024	−0.386	1	−0.345	−0.080	0.330	0.187	0.247
	*p*	0.252	0.921	0.102	0	0.148	0.744	0.167	0.444	0.308
SFCT	CC	−0.072	0.070	0.042	−0.345	1	−0.086	−0.048	0.142	0.027
	*p*	0.769	0.775	0.864	0.148	0	0.726	0.844	0.561	0.912
FRP	CC	−0.372	0.381	0.112	−0.080	−0.086	1	0.003	0.126	−0.144
	*p*	0.117	0.108	0.647	0.744	0.726	0	0.990	0.608	0.556
CCP	CC	−0.118	−0.084	−0.389	0.330	−0.048	0.003	1	−0.074	−0.308
	*p*	0.632	0.732	0.100	0.167	0.844	0.990	0	0.764	0.200
HLP	CC	0.221	0.000	−0.024	0.187	0.142	0.126	−0.074	1	0.231
	*p*	0.362	1.000	0.921	0.444	0.561	0.608	0.764	0	0.341
SLP	CC	0.440	−0.257	−0.252	0.247	0.027	−0.144	−0.308	0.231	1
	*p*	0.059	0.289	0.298	0.308	0.912	0.556	0.200	0.341	0

BCVA: best-corrected visual acuity; CC: correlation coefficient; CCP: choriocapillaris perfusion; CRT: central retinal thickness, duration: duration of silicone oil tamponade; FRP: full retinal perfusion; on/off: macular status preoperatively (whether macula was attached or detached); HLP: Haller’s layer perfusion; *p*: *p*-value; SFCT: subfoveal choroidal thickness; SLP: Sattler’s layer perfusion; TMV: total macular volume.

## Data Availability

Original datasets can be found at the department of ophthalmology, university clinic Lübeck.
